# Editorial: Trauma-Induced, DAMP-Mediated Remote Organ Injury, and Immunosuppression in the Acutely Ill Patient

**DOI:** 10.3389/fimmu.2019.01971

**Published:** 2019-08-20

**Authors:** Julien Pottecher, Alain Meyer, Camilla Ferreira Wenceslau, Kim Timmermans, Carl Jeffrey Hauser, Walter Gottlieb Land

**Affiliations:** ^1^Hôpitaux Universitaires de Strasbourg, Hôpital de Hautepierre, Service d'Anesthésie-Réanimation et Médecine Péri-Opératoire, Strasbourg, France; ^2^Université de Strasbourg, Faculté de Médecine, Fédération de Médecine Translationnelle de Strasbourg (FMTS), FRU 6702, EA3072, Strasbourg, France; ^3^Hôpitaux Universitaires de Strasbourg, Nouvel Hôpital Civil, Service de Physiologie et d'Explorations Fonctionnelles - Hôpital de Hautepierre, Centre de Référence National des Maladies Auto-immunes Rares, Service de Rhumatologie, Strasbourg, France; ^4^Université de Strasbourg, Faculté de Médecine, Fédération de Médecine Translationnelle de Strasbourg (FMTS), FRU 6702, EA3072, Strasbourg, France; ^5^Department of Physiology and Pharmacology, The University of Toledo College of Medicine and Life Sciences, Toledo, OH, United States; ^6^Radboud University Medical Center, Radboudumc Health Academy, Nijmegen, Netherlands; ^7^Acute Care Surgery and Critical Care, Beth Israel Deaconess Medical Center, Harvard Medical School, Boston, MA, United States; ^8^Université de Strasbourg, Faculté de Médecine, Fédération de Médecine Translationnelle de Strasbourg (FMTS), FRU 6702, Laboratoire d'Excellence Transplantex, Unité INSERM UMR_S1109, Immuno-Rhumatologie Moléculaire, Strasbourg, France

**Keywords:** trauma, damage-associated molecular pattern (DAMP), organ injury, Immunosuppression, critical care

Trauma is the third lead cause of mortality worldwide and is the first cause of fatality and invalidity in the 16–45 age group (1, 2). While early mortality is mainly due to overwhelming hemorrhage and catastrophic central nervous system injuries, later deaths are triggered by multi-organ failure and healthcare-acquired infections in patients demonstrating profound immunosuppression (3). While early deaths are uninterruptedly reduced along with road safety and pre-hospital care improvements, multi-organ failure and healthcare-acquired infections remain a serious burden for severe trauma patients (4). Indeed, 45% of patients admitted to a Level 1 Trauma Center develop multi-organ failure and infection remains the leading cause of death after trauma (5).

It is now thought that the innate immune system plays a key role in both trauma-induced remote organ failure and in trauma-induced immunosuppression. Indeed, according to the danger/injury model in immunology (6, 7), damage-associated molecular patterns (DAMPs) are massively released following severe musculoskeletal injury, which then bind to various receptors on the surface of and within neutrophils and elicit widespread systemic inflammation (8). As underscored by Meyer et al., mitochondria play a key role among DAMPs in polarizing the fate of inflammatory response. Indeed, mitochondrial DNA (mtDNA) has conserved unmethylated CpG motifs, characteristic of bacterial DNA, which are recognized by Toll-like receptor 9 (TLR9). Mitochondria also express “endogenous” N-formyl peptides, very close to bacterial formyl peptides, which both bind formyl peptide receptors on the surface of neutrophils. As such, pathogen-associated molecular patterns (PAMPs) and DAMPs share common cellular pathways driving indistinguishable clinical features. The polarization of the adaptive immune response involving mitochondria depends on the glycolytic/oxidative potential of the immune synapse.

In their review, Vourc'h et al. stress the biological importance of DAMPs as biomarkers for patient stratification, potential therapeutic targets and witnesses of immunological scar associated with severe trauma. To be clinically relevant, the ideal DAMP-biomarker should be immunologically active, its plasma concentration should reflect the severity of trauma and the extent of the inflammatory response. High Mobility Group Box One (HMGB1) is another redox-sensitive DAMP, which promotes Th2 immunity by favoring the proliferation of suppressive cells and the expansion of hypoactive monocytes. Besides extracellular compounds, intracellular multiprotein complexes (named inflammasomes) drive microcirculatory dysfunction, tissue injury and monocyte deactivation after traumatic brain injury and multiple trauma (Bortolotti et al.). After assembly, the three main inflammasomes (NLRP3, NLRP1, and AIM2) elicit the release of mature forms of Il-1ß, Il-18, and HMGB1, which may then trigger both a specific pro-inflammatory pyroptosis and a dysregulated monocyte function, paving the way to immunosuppression.

Remote hyperinflammation following trauma also impairs fracture healing. Indeed, activated neutrophils release their cytotoxic armamentarium and inhibit the synthesis of mineralized extracellular matrix by bone marrow stromal cells within the very first days after severe trauma (Bastian et al.). Both the number of bone marrow stromal cells and their osteogenic activity decrease when co-cultured with high neutrophil concentrations *in vitro*. Many indices converge on a close association between injury severity and the amount of DAMPs released. At the same time, DAMPs profoundly reduce innate and acquired immune responses. For instance, leukocyte HLA-DR gene expression and *ex-vivo* stimulated cytokine production negatively correlate with plasma levels of nuclear DAMPs released in human plasma after severe trauma (9).

Major surgical procedures such as cytoreductive surgery combined with hyperthermic intraperitoneal chemotherapy (CRS-HIPEC) are another source of controlled tissue trauma triggering the release of DAMPs and subsequent immunosuppression (Leijte et al.). After CRS-HIPEC, the increase in HMGB1 blood concentrations correlates with a decrease in HLA-DR expression and peaks at higher levels in patients who subsequently develop post-operative infections. DAMPs and extracellular vesicles may also drive remote thrombotic complications after trauma or surgical insults. Accurate characterization of DAMPs release and their consequences after trauma has broad clinical applications since it could entail an individualized approach for both preventive and curative therapeutic strategies. In this concept theory of secondary organ failures reviewed by Eppensteiner et al., many subcellular compounds (cell-free DNA, histones, S100 proteins, neutrophil extracellular traps, and microvesicles) drive remote thrombotic and inflammatory insults in organs distant from the primary insult. As such, they represent both diagnostic biomarkers of and potential therapeutic targets for the prevention of secondary organ failure.

Targeting those molecules upstream their binding to Toll-like receptors and pattern recognition receptors may be more therapeutically effective than trying to block their downstream pathways. For instance, membranes immobilized with nucleic acid-binding polymers, like hexadimethrine bromide, represent promising breakthroughs to dampen trauma-induced inflammation and remote organ injury, as exemplified by Aswani et al. In their clinical cohort of severe trauma patients, the authors show that patients who went on to develop secondary organ failures (acute lung injury in 93% of cases) had higher blood concentrations of mtDNA at only 2 h after injury. This mtDNA release may be triggered by both the mechanical tissue insult and the cellular injury instigated by shock-induced hypoperfusion. In a relevant animal model of severe trauma and shock, Aswani et al. subsequently showed that treatment with hexadimethrine bromide reduced both circulating levels of mtDNA and lung tissue injury (histological score).

Endothelium barrier breakdown, responsible for capillary leak, tissue hypoperfusion and vasoplegia is the mainstay of secondary organ failure, driven either by infective microorganisms or major trauma. In both situations, PAMPs and DAMPs, respectively bind formyl peptide receptors on non-immune cells (endothelial cells and vascular smooth muscle cells), which triggers cytoskeletal rearrangement, myogenic vascular contraction ending in endothelial barrier dysfunction. In their Hypothesis and Theory article, Martinez-Quinones et al. suggest that the breakdown of N-formyl peptide with deformylase (the degrading enzyme for N-formyl peptide) would safely reduce endothelium barrier breakdown after infective or mechanical tissue injury. Indeed, as opposed to a potential imbalance of transcriptional regulation associated with the inhibition of formyl peptide receptor, degrading N-formyl peptide would dampen the overwhelming inflammatory reaction without significant side effects.

In this Research Topic, we aim to shed light on DAMPs and the role they play in the interactive crosstalk between musculoskeletal trauma, remote organ injury and immunosuppression.

## Author Contributions

JP wrote, proofread, and approved the manuscript. AM, CW, KT, CH, and WL made substantial scientific content proofread and approved the manuscript.

### Conflict of Interest Statement

The authors declare that the research was conducted in the absence of any commercial or financial relationships that could be construed as a potential conflict of interest.
